# Clinical and microbial characterization of toxigenic *Clostridium difficile* isolated from antibiotic associated diarrhea in Egypt

**DOI:** 10.18502/ijm.v12i4.3932

**Published:** 2020-08

**Authors:** Sherein G. Elgendy, Sherine A. Aly, Rawhia Fathy, Enas A.E. Deaf, Naglaa H. Abu Faddan, Muhamad R. Abdel Hameed

**Affiliations:** 1 Department of Medical Microbiology and Immunology, School of Medicine, Assiut University, Assiut, Egypt; 2 Department of Pediatric Medicine, Assiut University Hospitals, Assiut University, Assiut, Egypt; 3 Department of Internal Medicine, Hematology Unit, Assiut University Hospitals, Assiut University, Assiut, Egypt

**Keywords:** Risk factor, *Clostridium difficile*, Toxigenic culture, Toxin genes

## Abstract

**Background and Objectives:**

*Clostridium difficile* infection (CDI) has become a significant healthcare-associated infection throughout the world and is particularly important in developing countries. This study aimed to investigate clinical characterization and risk factors related to toxigenic *C. difficile* infection in adult and pediatric patients, antimicrobial susceptibility pattern. Also, to evaluate different diagnostic methods for rapid detection of *C. difficile* associated diarrhea (CDAD) in Egypt.

**Materials and Methods:**

Stool samples were collected from 95 pediatric patients and 37 adult patients suffering from antibiotic associated diarrhea and were subjected to direct toxin immunoassay and culture on cycloserine/cefoxitin/fructose agar. The presence of *tcdA* and *tcdB* genes was tested by PCR.

**Results:**

Toxigenic *C. difficile* was isolated from pediatric and adult patients at a rate of 17.89% (17/95) and 27% (10/37) respectively. The sensitivity and specificity of direct PCR from stool are (100%, 100% and 82.4%, 100%) in adult and pediatric samples respectively. The susceptibility of *C. difficile* to vancomycin and metronidazole were found to be 66.7% and 48.2% respectively.

**Conclusion:**

Diabetes mellitus, prior antibiotic treatment, hematological malignancy on chemotherapy, malnutrition, neutropenia and Ryle feeding are risk factors for development of CDAD. Tight restriction of unnecessary antibiotic uses is necessary in our locality. Direct detection of toxin genes in stool by PCR is sensitive and specific method for early detection of *C. difficile.*

## INTRODUCTION

*Clostridium difficile* (CD) is widely distributed in human and animal feces. *C. difficile* associated diarrhea (CDAD) is a common cause of intestinal infection in hospital patients, usually starts 3 to 7 days following antibiotic administration and accounts for 10–25% of cases of antibiotic associated diarrhea (AAD) (1).

The main symptoms of *C. difficile* infection are fever, abdominal pain, diarrhea and severe pseudo-membranous colitis (PMC). Infection may lead to severe complications such as toxic megacolon and intestinal perforation, which is fatal (2).

The pathogenicity of *C. difficile* is based on the action of at least 1 of the 2 main toxins (A and B). After binding to appropriate receptors, toxins A and B are internalized and act on glucosyl transferases that modify guanose triphosphatases (GTPase) of the Rho and Ras families within the intestinal epithelial cells and lead to the disruption of the filamentous actin (F-actin) cytoskeleton. This is followed by dis-aggregation of polymerized actin, opening of tight junctions between cells, cell rounding and subsequent cell death. Molecular studies have determined that toxin A is encoded by the 8.1 kb *tcdA* gene, while toxin B is encoded by the 7.9 kb *tcdB* gene. Toxigenic strains of *C. difficile* possess this PaLoc, while non-toxigenic strains lack PaLoc (3).

Center for Disease Control and Prevention reports categorized *C. difficile* as an urgent threat. Antibiotic treatment for *C. difficile* infection (CDI) is often followed by recurrent infection, so nontraditional treatments, such as fecal transplant and oral administration of non-toxigenic *C. difficile* spores is needed (4). Significant patient related risk factors for CDI are antibiotic exposure, older age and hospitalization. Nearly every antibiotic has been associated with the development of CDI, including the drugs used for treatment of CDI (5).

Early detection of CDI and its toxins is critical to allow earlier treatment that can significantly reduce the morbidity, mortality, medical cost and family burden of CDI. The Food and Drug Administration (FDA) has approved a number of laboratory tests for the diagnosis of CDI including toxinogenic culture (TC), cell cytotoxicity neutralization assay (CCNA), enzyme immunoassays (EIA) for toxins A, B, and/or glutamate dehydrogenase (GDH), and nucleic acid amplification tests (NAATs) (6).

Nowadays, routine detection of *C. difficile* is not carried out in most hospitals in Egypt; as a result, missed diagnosis and delayed treatment may occur. There is no relevant detection method or clinical data in Assiut University hospitals, Egypt. This study aimed to investigate clinical characterization and risk factors related to toxigenic *C. difficile* infection in adult and pediatric patients, antimicrobial susceptibility pattern, also to evaluate the different diagnostic assays for the rapid detection of CDAD in Egypt.

## MATERIALS AND METHODS

### Ethical statement.

The Ethics Committee of the Faculty of Medicine, Assiut University, Egypt approved the study and informed consent was obtained from the participants or their parents.

### Patient population.

The study conducted in accordance with the clinical practice guidelines for *C. difficile* infection in adults and children updated by the Infectious Diseases Society of America (IDSA) and the Society for Healthcare Epidemiology of America (SHEA) (7).

This study included 2 groups of patients; adult and pediatric. Adult patients were 37 selected inpatients suspected to have AAD admitted to the Internal medicine & hematology ICU, Assiut University Hospitals. Pediatric samples were 95 and taken from children admitted to Gastroenterology and Hepatology unit of Assiut University children’s Hospital. For all selected patients, clinical and demographic data were included: age, hospital ward, date of hospital admission, medical condition, type and duration of antibiotic administration, non-surgical gastrointestinal procedures, anti-ulcer medications, chemotherapy for hematological malignancies, immunosuppressive therapy like those for aplastic anemia and other types of medications (8).

### Specimens.

Stool samples were collected in clean dry leak proof containers and sent to the laboratory within 1 hour. Stool samples were subjected to physical evaluation before processing. Each stool specimen was then divided into three aliquots, the first part was cultured immediately, the second part was tested for *C. difficile* Toxin A by EIA; the third part was frozen at −70°C for direct PCR testing. Routine bacteriological stool culture was performed to exclude the presence of other enteric pathogens, e.g. *Shigella, Salmonella*.

### Direct toxin detection from stool by EIA (9).

Enzyme immunoassay (Oxoid, UK), was used according to the manufacturer instructions. Briefly, 100 μl of stool sample was added to 1 ml of sample diluent in an eppendorf tube, vortex and centrifugation. Then 200 μl of the supernatant was transferred into the sample window of test card. Positive samples for toxin A production were suggested by the appearance of a detectable blue color in the result and control windows within 30 minutes. Negative samples were characterized by the presence of detectable blue color in the control window only.

### Culture and identification of *C. difficile* isolates.

Stool samples were first treated with absolute alcohol (alcohol shock) before inoculation on the selective medium to improve the selectivity of the medium (10). Equal volumes of stool and absolute ethanol were mixed and incubated at room temp for 1 or 2 hrs. Stool samples were then cultured on cycloserine-cefoxitin fructose agar (CCFA) (Oxoid, UK). The medium consists of animal peptones and fructose and is supplemented with D-cycloserine (500 μg/ml) and cefoxitin (16 μg/ml) that inhibit the growth of most normal fecal flora. The inoculated plates were incubated in an anaerobic jar using anaerogen gas packs (90% N2 /10% CO2) (Oxoid, UK), for 48–72 hours at 37°C.

Most strains of *C. difficile* when growing on CCFA medium exhibit a characteristic yellow, ground-glass colonial morphology. The cultured plates were examined under long-wave UV light. Suspected *C. difficile* colonies (based on colony morphology, Gram staining and the presence of yellowish-green fluorescence under long-wave UV light) were examined by Gram-staining and confirmed as *C. difficile* by latex slide agglutination test (Oxoid, UK). Suspected colonies were tested for their biochemical reaction profile reactions using API 20A for anaerobic bacteria (BioMerieux, France) according to the manufacturer instructions. Positive *C. difficile* isolates were further tested for toxin production by PCR amplification of the toxin genes (*tcdA* and *tcdB*) using DNA extracted from *C. difficile* colonies.

### PCR detection of *C. difficile* toxin genes.

DNA was extracted directly from stool samples as well as from *C. difficile* colonies. Direct extraction of DNA from stool samples was performed using the QIAamp DNA Stool Mini Kit (Qiagen, USA). Water boiling method was used for bacterial DNA extraction (11). PCR amplification of *C. difficile* toxin genes *(tcdA, tcdB)* and housekeeping gene *(tpi)* was performed according to the method described by Lemee et al. 2004 (11). The sequences of primers used in PCR amplification are listed in Table (1). DNA amplification was carried out in a Gene Amp9600 thermal cycler under the following conditions: initial denaturation for 5 minutes at 95°C, followed by a touchdown protocol consisting of 11 cycles of denaturation at 95°C for 30 s, annealing at temperatures decreasing from 65 to 55°C (with 1°C decremented steps in cycles 1 to 11) for 30 s, and DNA extension at 72°C for 60 s, this was followed by 29 cycles of denaturation at 95°C for 30 s, annealing at 55°C for 30 s, and extension at 72°C for 60 s, and lastly a final extension step at 72°C for 10 minutes. PCR products were visualized by electrophoresis on a 2% agarose gel stained with ethidium bromide.

**Table 1.** Primers used in PCR for molecular characterization of *C. difficile*, (Lemee et al. 2004)

**Gene target**	**Primer pair**	**Sequence (5′-3′)**	**Amplicon size (bp)**
*Tpi*	*tpi*-F	5′-AAAGAAGCTACTAAGGGTACAAA-3′	230
	*tpi*-R	5′-CATAATATTGGGTCTATTCCTAC-3	
*TcdA*	*tcdA*-F	5′-AGATTCCTATATTTACATGACAATAT-3′	369
	*tcdA*-R	5′-GTATCAGGCATAAAGTAATATACTTT-3′	
*TcdB*	*tcdB*-F	5′-GGAAAAGAGAATGGTTTTATTAA-3′	160
	*tcdB*-R	5′-ATCTTTAGTTATAACTTTGACATCTTT-3	

### Antibiotic susceptibility testing.

Antibiotic susceptibility testing was performed according to the Clinical Laboratory Standard Institute (CLSI) guidelines using Kirby-Bauer method (CLSI, 2014) (12). Antibiotics used were benzyl penicillin (10U), piperacillin tazobactam (100-10 μg), amoxicillin clavulanic acid (20-10 μg), impenem (10 μg), ceftriaxone (30 μg) chloramphenicol (30 μg), tetracycline (30 μg), moxifloxacin (5 μg), ciprofloxacin (5 μg), levofloxacin (5 μg) vancomycin (5 μg) and metronidazole (4 μg) (Oxoid, UK). Inoculated Mueller Hinton agar plates (HiMedia, India) were incubated at 37°C for 24 hours in the anaerobic jar using Anaerogen gas packs (90% N2 /10% CO2) (Oxoid, UK). *E. coli* ATCC 25922 was used as standard strain to check the standardization of the disks.

### Statistical analysis.

Data were statistically described in terms of the mean standard deviation (SD), median and range, or frequencies (number of cases) and percentages when appropriate. Comparison of numerical variables between the study groups was done using Mann Whitney U test for independent samples. For comparing categorical data, exact test was used instead when the expected frequency is less than 5. P values less than 0.05 was considered statistically significant. All statistical calculations were done using computer programs SPSS (Statistical Package for the Social Science; SPSS Inc., Chicago, IL, USA) version 15 for Microsoft Windows.

## RESULTS

### Toxigenic *C. difficile* pathogens.

According to the results of the anaerobic stool culture and API biochemical profile, *C. difficile* pathogens were isolated from (24/95) pediatric stool samples and (10/37) adult stool samples. All recovered *C. difficile* isolates were confirmed by latex agglutination test and PCR amplification of the housekeeping gene *(tpi)*. Following toxigenic culture (anaerobic culture followed by PCR amplification of toxin gene from bacterial DNA), (17/24) of pediatric *C. difficile* isolates and (10/10) of adult *C. difficile* isolates were found to be toxigenic.

### Evaluation of different toxigenic *C. difficile* diagnostic methods.

**Fig. 1.**
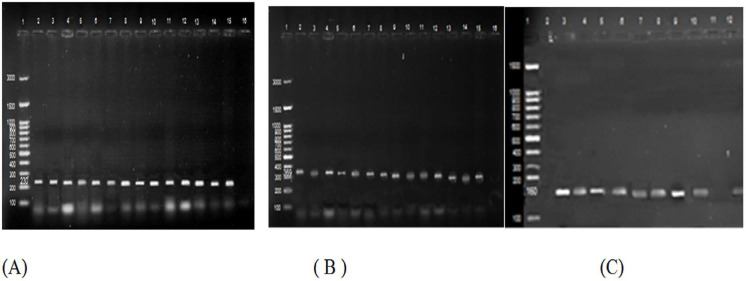
Gel electrophoresis (A) Detection of *tpi* gene at 230 bp; Lane 1: DNA molecular weight marker, Lanes (2–15) positive samples. Lane 16: negative isolate. (B) Detection of *tcdA* gene at 369 bp; Lane 1: DNA molecular weight marker, Lanes (2–15) positive samples. Lane 16: negative isolate. (C) Detection of *tcdB* gene at 160 bp; Lane 1: DNA molecular weight marker, Lanes (3–10) positive samples, Lane 2, 11: negative isolates.

Regarding adult stool samples, toxigenic stool culture found out that the10 *C. difficile* isolates identified by the anaerobic stool culture and the API biochemical profiles are toxigenic. Toxin A immunoassay detected 11 toxigenic *C. difficile* isolates identifying an additional toxin producing non *C. difficile* isolate. The direct PCR from stool samples identified 10 *C. difficile* isolates by detecting tpi and all of them were found toxigenic because of presence of both *tcdA* and *tcdB* (Fig. 1). Considering the toxigenic stool culture as the “standard”, the sensitivities, specificities, positive and negative predictive values, and accuracies of the assays, respectively, were (100%, 96.3%, 90.9%, 100% and 97.3%) for direct toxin A immunoassay; and (100%, 100%, 100%, 100% and 100%) for direct PCR assay.

Concerning the 24 *C. difficile* isolates recovered from pediatric stool samples, (17/24) isolates were found to be toxigenic by the toxigenic stool culture assay. Toxin A immunoassay detected 25 toxigenic *C. difficile* isolates detecting toxin production from 17 toxigenic *C. difficile* isolates and one of the non-*C. difficile* isolate. The direct PCR targeting *tpi* from stool samples only identified 21 *C. difficile* isolates with 14 of them were toxigenic as they contained both *tcdA* and *tcdB*. However, 3 toxigenic *C. difficile* isolates (detected by toxigenic culture) were negative for the three genes. The sensitivities, specificities, positive and negative predictive values, and accuracies of the assays, respectively, were (100%, 89.7%, 68%, 100%, 91.6%) for direct toxin A immunoassay; (82.4%, 100%, 100%, 96.1% and 96.7%) for direct PCR assay.

### Risk factors in adult patients.

A cohort of 37 patients was used to describe risk factors associated with acquiring toxigenic *C. difficile* diarrhea, Table (2). Diabetic patients and Patients with previous antibiotic therapy, neutropenia and hematological malignancy on chemotherapy were at a higher risk for acquiring *C. difficile* with statistically significant difference (P < 0.05). Patients taking antacid and Ryle feeding were at higher risk for acquiring *C. difficile* with statistically highly significant difference (P < 0.001). Urinary catheterization did not differ statistically among patients who acquired *C. difficile* compared with those who did not (P > 0.05).

**Table 2.** Risk factors for CDAD in adult ICU

**Risk factor**	**Total No. of patients (37)**	**No. of *C. difficile* (10)**	**% of infection**	**R.R.**	**O.R.**	**P. value**
Diabetes						
Yes	12	5	41.7	2.08	2.86	<0.05^*^
No	25	5	20			
Antibiotic treatment						
Yes	26	9	38.5	3.81	5.29	<0.05^*^
No	11	1	9.00			
Sex						
Females	10	3	30	1.15	1.22	<0.05^*^
Males	27	7	25.9			
Malnutrition						
Yes	11	5	45.5	2.36	3.50	<0.05^*^
No	26	5	19			
Hematological malignancy on chemotherapy						
Yes	12	6	50	3.12	5.25	<0.05^*^
No	25	4	16			
Neutropenia (Aplastic anemia induced)						
Yes	9	3	33.3	1.33	1.50	<0.05^*^
No	28	7	25			
Antacid intake						
Yes	27	9	33.3	3.33	4.50	0.001^**^
No	10	1	10			
Ryle feeding						
Yes	8	7	87.5	8.45	60.67	0.001^**^
No	29	3	10			
Urinary catheterization						
Yes	17	4	23.5	0.78	0.718	>0.05
No	20	6	30			

RR: Relative risk, O.R. Odds ratio,

* P values less than 0.05 was considered statistically significant,

** P values = 0.001 was considered statistically highly significant

### Risk factors in pediatric patients.

A cohort of 95 patients was used to describe risk factors associated with acquiring toxigenic *C. difficile* diarrhea, Table (3). Patients suffering from recurrent diarrhea, neutropenia and Ryle feeding were at a higher risk for acquiring *C. difficile* with statistically highly significant difference (P < 0.001).

**Table 3.** Risk factors for CDAD in children

**Risk factor**	**Total No. of patients (95)**	**No. of *C. difficile* (17)**	**% of infection**	**R.R.**	**O.R.**	**P. value**
Recurrence						
Yes	19	11	58	7.33	16.04	0.001^**^
No	76	6	7.9			
Ryle feeding						
Yes	5	4	80	5.538	23.69	0.001^**^
No	90	13	14			
Antacid intake						
Yes	64	16	25	7.75	10.00	<0.05^*^
No	31	1	3			
Neutropenia						
Yes	23	14	60	14.61	35.78	0.001^**^
No	72	3	4			
ICU admission						
Yes	10	7	70	5.93	17.5	0.001^**^
No	85	10	11.7			

RR: Relative risk, O.R. Odds ratio,

* P values less than 0.05 was considered statistically significant,

** P values = 0.001 was considered statistically highly significant

### Antimicrobial susceptibility.

Antibiotic sensitivity of toxigenic *C. difficile* showed that isolates were mostly sensitive to moxifloxacin (74.1%), levofloxacin (70.4%), Metronidazole (48.2%), vancomycin (66.7%) and impenem (62.9%) and mostly resistant to ceftriaxone (66.7%) and piperacillin/tazobactam (55.6%), (Table 4).

**Table 4.** Antimicrobial susceptibility of toxigenic *C. difficile* isolates

**Antibiotic Total used isolates (27)**	**S**		**I**		**R**	

	**No.**	**%**	**No.**	**%**	**No.**	**%**
Benzyl penicillin	13	48.2	2	7.4	12	44.4
Piperacillin / tazobactam	8	29.6	4	14.8	15	55.6
Amoxicillin Clavulanic acid	10	37.0	7	26.0	10	37.0
Ceftriaxone	7	25.9	2	7.4	18	66.7
Vancomycin	18	66.7	8	29.6	1	3.7
Ciprofloxacin	12	44.4	7	26.0	8	29.6
Moxifloxacin	20	74.1	2	7.4	5	18.5
Levofloxacin	19	70.4	2	7.4	6	22.2
Tetracycline	7	25.9	2	7.4	18	66.7
Chloramphenicol	11	40.7	1	3.7	15	55.6
Impenem	17	62.9	3	11.1	7	26.0
Metronidazole	13	48.2	1	3.6	13	48.2

No = number of samples, % = percentage is calculated according to number of samples obtained

## DISCUSSION

*C. difficile* associated diarrhea (CDAD) has emerged as a major public health problem. Outbreaks of CDAD have been described in many countries such as Iran (9), Germany (13), France (14) and Canada (15). *C. difficile* is increasingly being recognized as an important pediatric enteric pathogen in healthcare and community settings, particularly in children 1–5 years of age, including children without traditional risk factors for *C. difficile* infections (16).

Studies on *C. difficile*-associated diarrhea in Egypt are limited, probably due to the lack of technology and facilities for the culture and identification of anaerobic pathogens. In this study *C. difficile* were isolated from pediatric patients at a rate of 25.2% (24/95), while in adult patients at a rate of 27% (10/37). These results are in parallel to previous studies in Egypt where Helim and Hamdy, 2006 (17) reported an isolation rate of *C. difficile* of (35%) in Kasr Al-Aini hospital, Cairo University. Other study reported that the isolation rate of CDAD ranged from 25% to 30% among patients suffering from diarrhea (18). However, lower rates of *C. difficile* isolation were reported in Brazil (5.5%) (8), UK (1.52 to 4.78%) (19), Iran (6.1%) (20), Saudi Arabia (9%) (21) and Jordan (13.7%) (22). In a previous Egyptian study, the rate of *C. difficile* isolation was (1.3% and 2%) which is much lower than that reported in this present study (23). The high *C. difficile* rate in our community may be attributed to the indiscriminate use of antibiotics in our locality. In addition, the alcohol shock treatment before inoculation into solid media might explain the higher isolation rate than other Egyptian studies.

Zhong Peng et al. (2018) reported that preliminary treatment with “heat shock” or “alcohol shock” in order to recover *C. difficile* from stool specimens will minimize the contaminating growth of other stool organisms (24).

Elnaze Zare Mirzaei et al. (2018) reported that diagnosis of *C. difficile* associated diarrhea can be achieved by a number of techniques including culture on anaerobic media, cell cytotoxicity and PCR. Toxigenic culture which includes anaerobic culture on CCFA, biochemical reaction followed by either PCR amplification of toxin genes or cell cytotoxicity is considered the standard for diagnosis of CDAD (9). Although toxigenic stool culture is the most sensitive test and hence considered the “gold standard” for detecting *C. difficile*, but its acceptance is limited due to its slow turnaround time (25).

Based on our aim to identify a relevant but rapid technique for detection of *C. difficile* in patients with diarrhea, we performed a rapid direct Toxin A Enzyme Immunoassay on stool samples and a direct PCR for detection of *tcdA* and *tcdB* in addition to the standard toxigenic stool culture method. Our results show that toxigenic *C. difficile* was isolated from pediatric and adult patients at a rate of 17.89% (17/95) and 27% (10/37) respectively. The Direct PCR is considered a sensitive and specific (100%, 100% and 82.4%, 100%) for the detection of toxigenic *C. difficile* from stool samples both in adult and pediatric patients. Direct PCR failed to detect toxigenic *C. difficile* from 3 pediatric stool samples, which could be explained by the solid consistency of these samples that hindered DNA extraction. On the other hand, direct toxin A immunoassay although found sensitive but with lower specificity, positive values, and accuracy. These results are in agreement with what have been previously published reports (9, 24).

Diabetes was found to comprise risk for occurrence of CDAD (RR: 2.08, OR = 2.86, P < 0.05) which was in accordance with what has been reported by Hui-Qi and Jiang (26) (OR: 2.99 and p < 0.05). Patients under antibiotic treatment were at a higher risk for acquiring *C. difficile* (RR: 3.81, OR: 5.29, p <0.05). These results are in agreement with those of Gianni et al. (27) who reported that previous antibiotic use is the predominant risk factor for *C. difficile* acquisition, with relative risk of 5.9. Females were at a significantly higher risk for acquiring *C. difficile* than males (RR: 1.5, OR: 1.22, p < 0.05) which agreed with what was reported by Timothy et al. (28) (OR: 1.50, p: < 0.05). Patients who received proton pump inhibitors (PPI) were found to be at a higher risk for the development of CDAD (RR: 3.33, OR: 4.50, P = 0.001) which is in accordance with what was demonstrated by Aseeri et al. (29) who found that CDAD was associated with the use of PPI (OR = 3.6, p = 0.001). Sahil and Darrell. (30) reported that malnutrition, immunosuppression, neutropenia and Ryle feeding play an important role in acquisition of CDAD which is similar to the results reported in this study. On the contrary, urinary catheterization did not differ statistically among patients who acquired *C. difficile* compared with those who did not (P > 0.05) and these observations agreed with what was reported by Aseeri et al. (28). Similarly, we found that recurrent diarrhea, Ryle feeding, antacids in-take, neutropenia and antibiotic administration have been shown to play an important role in acquiring pediatric CDAD. These results are in parallel to those reported by previous studies (30, 31).

The disk diffusion method seems to be a good method to detect *C. difficile* isolates suspected to have a decreased susceptibility (32). Results of antimicrobial susceptibility testing of *C. difficile* showed that the isolates were mostly sensitive to moxifloxacin (74.1%), levofloxacin (70.4%), vancomycin (66.7%) and impenem (62.9%) and mostly resistant to ceftriaxone (66.7%) and piperacillin /tazobactam (55.6%). Similar results have been reported by Poilane et al. (32) who found that resistance to piperacillin /tazobactam (60%) and to ceftriaxone (100%).

Fluoroquinolones reported to have a good activity against Gram-positive bacilli including *C. difficile*. However, higher rates of resistance to different classes of fluoroquinolones have emerged worldwide (33). In the present study, *C. difficile* isolates were found to be sensitive to moxifloxacin (74.1%) and levofloxacin (66.7%).

Although in most of the studies investigating the antimicrobial susceptibility of *C. difficile*, decreased susceptibility to vancomycin among *C. difficile* has been reported, but it is still used as an effective drug for treatment of CDAD (34). In the present study, the susceptibility of *C. difficile* to vancomycin was found to be (66.7%). In the contrary, Poilane et al. (32) and El-Sokkary et al. (35) reported (0%) resistance for vancomycin. This difference is duo to lacking of good antibiotic policy in our locality and no tight restriction of unnecessary antibiotic uses.

Previous studies reported that metronidazole or oral vancomycin remains the treatments of choice for patients with CDI; however, resistance to both agents is continuously increasing (36, 37). In the present study, the susceptibility of *C. difficile* isolates to metronidazole was found to be (48.2%) while El-Sokkary et al. (35) reported that only one strain was resistant to mertonidazole. The results of antibiotic susceptibility pattern of *C. difficile* isolates recovered in this study show that nearly half of the isolates are resistant to metronidazole and vancomycin which might lead to treatment failure of this pathogen in the near future. Therefore rationale use of antimicrobials is mandatory to prohibit further exaggeration of the problem.

## CONCLUSION

Toxigenic *C. difficile* represents an important etiologic agent of *C. difficile* associated diarrhea both in adult and pediatric patients at Assiut University Hospitals. Diabetes mellitus, antibiotic treatment, hematological malignancy on chemotherapy, malnutrition, neutropenia, antacid intake and Ryle feeding are risk factors for development of adult and pediatric CDAD. Nearly half of the isolates are resistant to metronidazole and vancomycin, therefore rationale use of antimicrobials is mandatory to prohibit further exaggeration of the problem. Although direct detection of *C. difficile* genes from stool samples based on PCR is expensive, yet this method is more sensitive and less time-consuming than culture methods and provides greater sensitivity than an enzyme immunoassay.
